# Comparison of *Staphylococcus* Phage K with Close Phage Relatives Commonly Employed in Phage Therapeutics

**DOI:** 10.3390/antibiotics7020037

**Published:** 2018-04-25

**Authors:** Jude Ajuebor, Colin Buttimer, Sara Arroyo-Moreno, Nina Chanishvili, Emma M. Gabriel, Jim O’Mahony, Olivia McAuliffe, Horst Neve, Charles Franz, Aidan Coffey

**Affiliations:** 1 Department of Biological Sciences, Cork Institute of Technology, Bishopstown, Cork T12 P928, Ireland; jude.ajuebor@mycit.ie (J.A.); colin.buttimer@mycit.ie (C.B.); sara.arroyo-moreno@mycit.ie (S.A.-M.); emma.gabriel@mycit.ie (E.M.G.); Jim.OMahony@cit.ie (J.O.); 2 Eliava Institute of Bacteriophages, Microbiology and Virology, Tbilisi 0160, Georgia; nina.chanishvili@pha.ge; 3 Teagasc, Moorepark Food Research Centre, Fermoy, Cork P61 C996, Ireland; Olivia.McAuliffe@teagasc.ie; 4 Department of Microbiology and Biotechnology, Max Rubner-Institut, DE-24103 Kiel, Germany; horst.neve@mri.bund.de (H.N.); charles.franz@mri.bund.de (C.F.); 5 Alimentary Pharmabiotic Centre, University College, Cork T12 YT20, Ireland

**Keywords:** phage isolation, bacteriophage, phage resistance, MRSA, *Staphylococcus*, *Kayvirus*

## Abstract

The increase in antibiotic resistance in pathogenic bacteria is a public health danger requiring alternative treatment options, and this has led to renewed interest in phage therapy. In this respect, we describe the distinct host ranges of *Staphylococcus* phage K, and two other K-like phages against 23 isolates, including 21 methicillin-resistant *S. aureus* (MRSA) representative sequence types representing the Irish National MRSA Reference Laboratory collection. The two K-like phages were isolated from the *Fersisi* therapeutic phage mix from the Tbilisi Eliava Institute, and were designated B1 (vB_SauM_B1) and JA1 (vB_SauM_JA1). The sequence relatedness of B1 and JA1 to phage K was observed to be 95% and 94% respectively. In terms of host range on the 23 *Staphylococcus* isolates, B1 and JA1 infected 73.9% and 78.2% respectively, whereas K infected only 43.5%. Eleven open reading frames (ORFs) present in both phages B1 and JA1 but absent in phage K were identified by comparative genomic analysis. These ORFs were also found to be present in the genomes of phages (Team 1, vB_SauM-fRuSau02, Sb_1 and ISP) that are components of several commercial phage mixtures with reported wide host ranges. This is the first comparative study of therapeutic staphylococcal phages within the recently described genus *Kayvirus*.

## 1. Introduction

*Staphylococcus aureus* (*S. aureus*) is an opportunistic and important pathogen in clinical and health-care settings causing a wide variety of diseases commonly involving the skin, soft tissue, bone, and joints [1]. It is also a well-known causative agent of prosthetic joint infections (PJI), cardiac device infections, and intravascular catheter infections [1]. *S. aureus* pathogenicity is due, in part, to its ability to acquire and express a wide array of virulence factors, as well as antimicrobial resistance determinants [2], an example of which involves the acquisition of the staphylococcal cassette chromosome (SCCmec) leading to the development of methicillin resistance in *S. aureus* [3]. Methicillin-resistant *S. aureus* (MRSA) was first reported in 1961 [4], and has since been observed to cause serious infections in hospitals worldwide. Reports of MRSA clones resistant to the majority of antibiotics are a growing concern [5]. As such, new treatment options are needed.

Bacteriophages (phages) are biological entities composed of either DNA or RNA enclosed within a protein coat [6]. They are highly specific, with most phages capable of infecting only a single bacterial species [6,7], and studies on these viruses have been performed since the late 19th century [8]. The phage infection process usually begins with the recognition of the receptor on the bacterial cell surface by its receptor binding protein [9]. In natural environments bacterial hosts have evolved many mechanisms to protect themselves from phage attack to include; adsorption blocking, DNA injection blocking, restriction-modification system (R/M), abortive infection, and the clustered regularly interspaced short palindromic repeats (CRISPR)-Cas systems [10,11]. In turn, phages have evolved several strategies for overcoming these systems to ensure their survival in the phage-host co-evolutionary race [12,13,14].

The use of phages as therapeutics to eliminate pathogenic bacteria dates back to experiments conducted by Felix d'Herelle in 1919 at a French hospital to treat dysentery [15]. Since then, a wide range of phage therapy trials have been undertaken, many with very promising results [15,16]. Pyophage and Intesti-phage are among the commercial phage mixtures currently produced at the Eliava Institute. Metagenomic studies on these phage mixtures have been reported [17,18] and the staphylococcal phages Sb-1 and ISP are key components of Pyophage [19,20]. Other phages isolated from these commercial phages mixes have also been reported [21,22,23,24]. Phages like vB_SauM-fRuSau02 was isolated from a phage mix produced by Microgen (Moscow, Russia) [21] and Team 1 was isolated from PhageBioDerm, a wound healing preparation consisting of a biodegradable polymer impregnated with an antibiotic and lytic phages [22,23,24]. These phages all possess a wide host range against a number of clinically relevant *S. aureus* isolates, demonstrating the efficacy of such commercial phage mixtures in treating a range of bacterial infections [19,20,21,22,23,24].

In this paper, we employed another phage mixture from the Eliava Institute, namely the Fersisi phage mix. Fersisi is a relatively new combination developed approximately 15–20 years ago on the basis of Pyophage, although with fewer phage components. Two phages from this mix were designated B1 (vB_SauM_B1) and JA1 (vB_SauM_JA1). Phage K, on the other hand, is a well-known phage being the type phage of the recently designated genus *Kayvirus* of the subfamily *Spounavirinae* [25]. The exact origin of phage K is unknown, but descriptions of the phage are made as far back as 1949 [26,27]. An initial host range study involving this phage reported it to be ineffective against many MRSA strains [26]. Thus, phages B1 and JA1 were compared (on the basis of their host range) to phage K to explore possible host range differences and it was observed that both phages had broader host ranges. A comparative study was performed on their genomes and the genomes of similar phages from other commercial phage mixtures (Team 1, vB_SauM-fRuSau02, Sb_1 and ISP) with reported wide host ranges, to provide molecular insight into the differences in host range encountered in this study.

## 2. Results and Discussion

### 2.1. Origin of Phages B1 and JA1

Phages B1 and JA1 were isolated from the Fersisi commercial phage mixtures; batch 010112 (B1) and F-062015 (JA1). This product is used in the treatment of staphylococcal and streptococcal infections. For the isolation of B1, phage enrichment was carried out using staphylococcal host cultured from the sonicate fluid of a hospital patient suffering from PJI. DPC5246 was subsequently used as propagating host for B1, as a prophage was encountered in the PJI strain. Phage enrichment in the isolation of JA1 was done using the Cork Institute of Technology (CIT) collection strain *S. aureus* CIT281189. Both the PJI strain and CIT281189 were insensitive to phage K.

### 2.2. Morphology and Host Range of Phages K, B1 and JA1

**Figure 1:**
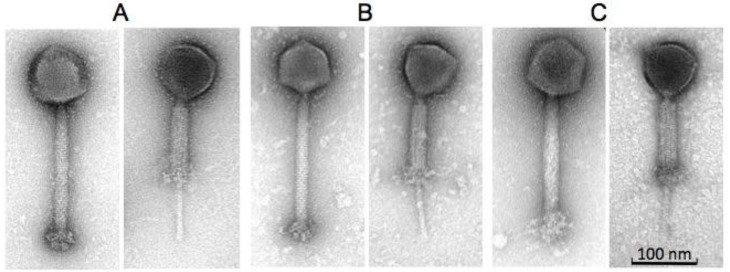
Transmission electron micrographs of phages B1 (**A**), JA1 (**B**), and K (**C**) showing their icosahedral capsid and their long contractile tail (both extended and contracted).

Phages B1 and JA1 exhibited typical characteristics of phages belonging to the *Myoviridae* family, similar to the reported morphology of phage K [26]. All three phages possessed an A1 morphology [28], displaying an icosahedral head as well as a long contractile tail. They also contained a structure previously described as knob-like appendages by O'Flaherty et al. [26], extending from their base plates (likely “clumped/aggregated” base plate appendices) and clearly visible in Figure 1. Estimations were made on the dimensions of these phages (Table 1). Capsid heights were estimated as 92.9 ± 4.0 nm (B1), 87.0 ± 2.1 nm (JA1) and 92.9 ± 3.8 nm (K). Tail dimension were also estimated as 233.0 ± 4.4 × 23.4 ± 1.2 nm (B1), 231.5 ± 4.7 × 22.7 ± 0.9 nm (JA1), and 227.5 ± 5.5 × 23.8 ± 1.0 nm (K), and base plates/knobs complexes were estimated as 30.1 ± 1.8 × 47.2 ± 3.7nm (B1), 32.5 ± 7.9 × 45.8 ± 1.4 nm (JA1), and 36.6 ± 5.1 × 41.7 ± 2.6 nm (K). Owing to the similar morphology of all three phages, a host range study was conducted to explore possible differences in host spectra across a number of hospital isolates. Twenty-one of these isolates represented the entire collection of MRSA sequence-types identified in Ireland by the National MRSA Reference Laboratory (Dublin, Ireland), and includes the commonly encountered ST22-MRSA-IV, which has been predominant in Irish hospitals since the late 1990s [29]. The other two *S. aureus* strains used in this study were included as additional phage propagation strains. Host range was assessed by plaque assay technique on lawns of various MRSA strains listed in Table 2. The efficiency of plaquing (EOP) was used to represent the degree to which each of the phages studied infected all 23 staphylococcal strains. Phage JA1 had the broadest host range, forming plaques on 18 out of the 23 staphylococcal strains examined. B1 also had a broad host range and was capable of forming plaques on 17 isolates (with some in common with the 18 lysed by phage JA1). Phage K had the narrowest host range, forming plaques on only 10 of the isolates (including its propagating strain DPC5246). All 23 staphylococcal strains were effectively lysed by at least one of the three phages, with the exception of E1139 (IV) ST45 and E1185 (IV) ST12, whose EOP were significantly low at 3.88 × 10^−6^ and 1.16 × 10^−6^ respectively; as well as 3488 (VV) ST8, which was resistant to all three phages. Plaque size ranged from 0.5 mm to 1.5 mm, with a halo occurring in some instances (Table 3 and Supplementary Materials, Figure S1). The wide host range encountered in this study is common among K-like phages and has been reported for other staphylococcal K-like phages, such as JD007, which infected 95% of *S. aureus* isolates obtained from several hospitals in Shanghai, China [30].

### 2.3. Phage Adsorption on Phage Resistant Isolates

While some level of phage insensitivity was encountered against all three phages, phage K was the frequently insensitive virion to the *S. aureus* strains tested, and thus, was chosen to evaluate whether or not adsorption inhibition played a role in its insensitivity. Phage K was able to adsorb to all phage-insensitive strains to approximately the same extent as the propagating strain DPC5246. This rules out the possibility of adsorption inhibition playing a role in the narrow host range encountered with phage K in comparison to both phages B1 and JA1 (Supplementary Materials, Figure S2). Additionally, adsorption studies with phages B1 and JA1 indicated that adsorption did not play a role in the differences observed.

### 2.4. Genome Comparison between Phages B1, JA1, and K

The genome of phage K is 139,831 bp in size with long terminal repeats (LTRs) of 8486 bp [31]. Genomes of similar sizes were obtained for phages B1 and JA1, these being 140,808 bp and 139,484 bp, respectively. Examination of sequence reads allowed the identification of LTRs for these phages, due to the identification of a region within their genomes with roughly double the average number of reads, these regions being 8076 bp and 7651 bp in size for phages B1 and JA1, respectively. This approach to the determination of terminal repeats has been utilized for a number of phages [32,33,34]. The sequences of all three phages, when analyzed, contained the 12 bp inverted repeat sequences 5′-TAAGTACCTGGG-3′ and 5′-CCCAGGTACTTA-3′, which separates the LTRs from the non-redundant part of the phage DNA, and are characteristic of K-like phages [22,35]. Thus, the entire packaged genome sizes are 148,884 bp (B1), 147,135 bp (JA1), and 148,317 bp (K). Phage K possessed 212 ORFs in its genome [31,36], whereas phages B1 and JA1 possessed 219 (Supplementary Materials, Table S1) and 215 ORFs (Supplementary Materials, Table S2) respectively.

Nucleotide pairwise sequence alignment based on BLASTN revealed phages B1 and JA1 (including their LTRs) to be 99% identical to each other, thus can be considered different isolates of the same phage species [37]. On the other hand, phages B1 and JA1 (including their LTRs) showed 95% and 94% identity (respectively) to phage K, placing these phages on the boundary of speciation.

The examination of 100 bp sequences upstream of each ORFs on the non-redundant genome of these phages, using MEME [38], identified 44 and 43 RpoD-like promoters for phages B1 and JA1, respectively. It was observed that these promoters where heavily concentrated in regions with ORFs encoding short hypothetical proteins and those with functions associated with nucleotide metabolism and DNA replication, rather than those associated with virion structure (Supplementary Materials, Tables S3 and S4). A similar finding was also reported with K-like phage vB_SauM-fRuSau02 [21]. Additionally, 30 Rho-independent terminators were identified on the non-redundant genomes for both B1 and JA1 (Supplementary Materials, Tables S5 and S6). 

**Figure 2:**
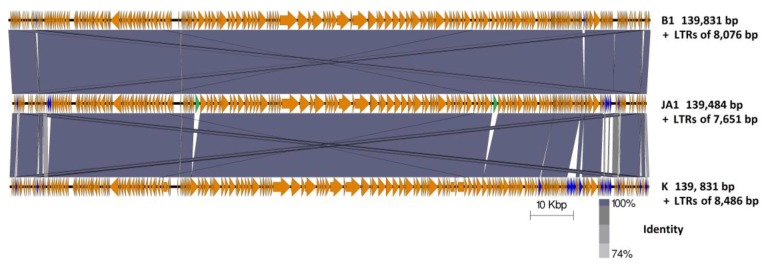
Genome comparison of phages B1, JA1, and K (including their long terminal repeats) using currently available annotations employing BLASTN and visualized with Easyfig. Regions of sequence similarity are connected by the shaded area, using a grey scale; genome maps consisting of orange arrows indicating the location of ORFs along the phage genomes, with unshared ORFs highlighted in blue with those indicating unshared homing endonuclease highlighted in green.

Four ORFs present in phage B1 were observed to be absent in JA1 (Table 4). These ORFs encoded two putative terminal repeat-encoded proteins (PhageB1_009, 016) and two proteins of unknown function (phageB1_202, 203). Although both B1 and JA1 had similar content of ORFs with 1% difference between their genomes, both phages varied in their host range on the *S. aureus* strains they infected. This variation is likely attributed to the difference encountered in their genome. Additionally, multiple ORFs present in phage K but absent in both B1 and JA1 were encountered (Figure 2, Table 5). Furthermore, ORFs present in both phages B1 and JA1 but absent in K were also encountered (Figure 2, Table 6). These ORFs are discussed below.

#### 2.4.1. Characteristic Features of Phage K ORFs Absent in Both JA1 and B1

Seventeen ORFs present in phage K were absent in both phages B1 and JA1, with one additional ORF found not to be shared between JA1 and K. These ORFs are listed in Table 5. No function could be assigned to these with the exception of phageK_190, which based on NCBI conserved domain search possessed a metallophosphatase-like domain (cd07390; E value; 3.94 × 10^−30^) and is a member of the metallophosphatase (MPP) superfamily. Families within this superfamily of enzymes are functionally diverse, involved in the cleavage of phosphoester bonds, and include Mre11/SbcD-like exonucleases, Dbr1-like RNA lariat debranching enzymes, YfcE-like phosphodiesterases, purple acid phosphatases (PAPs), YbbF-like UDP-2,3-diacylglucosamine hydrolases, and acid sphingomyelinases (ASMases) [39]. 

#### 2.4.2. Characteristic Features of Phages B1 and JA1 ORFs Absent in Phage K 

Eleven ORFs present in both phages B1 and JA1 were absent in phage K (Table 6). No putative function could be assigned to the majority of these ORFs based on BLASTP, InterProScan or HHpred analysis, with the exception of phageJA1_084 (phageB1_087) and phageJA1_152 (phageB1_155), which encoded homing endonucleases interrupting both the terminase large subunit and the DNA repair protein, respectively. These homing endonucleases are site-specific DNA endonucleases capable of initiating DNA breaks leading to repair and recombination event that results in the integration of this endonuclease ORF into a gene that was previously lacking it [40]. The presence of these mobile genetic elements is common among known staphylococcal phages of the subfamily *Spounavirinae*, and these endonucleases ORFs are known to insert themselves into essential phage genes [21,41]. Additionally, HHpred analysis indicated ORFs PhageJA1_208 and PhageB1_214 to possess remote homology to cell-degrading proteins. The majority of these ORFs were found to be located next to the genome termini of JA1 and B1, with genes located in this region having been previously reported in similar phages to be expressed early in phage development [35]. Such proteins are usually involved in subversion of the host’s machinery to aid phage takeover [42,43]. 

#### 2.4.3. Comparison of Phages K, B1, and JA1 with other Similar Therapeutic Phages (Team1, vB_SauM-fRuSau02, Sb-1 and ISP)

**Figure 3:**
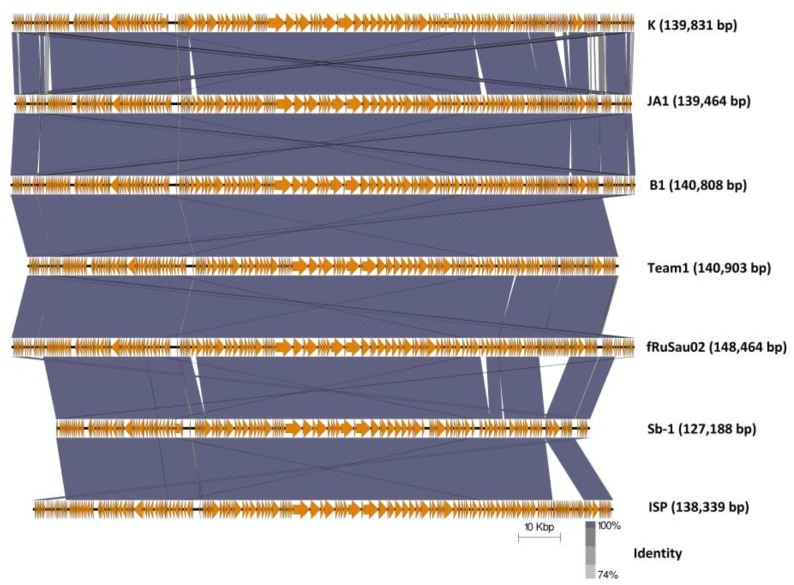
Genome comparison of phage K with the six staphylococcal phages employed in commercial phages mixture (B1, JA1, Team 1 [22,23,24], vB_SauM-fRuSau02 [21], Sb-1 [19] and ISP [20]) using currently available annotations employing BLASTN and visualized with Easyfig.

Four additional staphylococcal phages that originate in commercial phage therapeutic mixtures are Team1, vB_SauM-fRuSau02, Sb-1 and ISP, as discussed earlier [19,20,21,22,23,24]. These phages were also reported to possess wide host ranges towards a number of clinically relevant *S. aureus* strains. Although similar, these phages have several feature differences from each other and from phages B1 and JA1. Comparison of nucleotide identities (BLASTN) with phage K shows that they belong to the genus *Kayvirus* (Supplementary Materials, Table S7) possessing genomes of similar sizes, apart from Sb-1, being smaller than would be expected, suggesting the genome submission may have been incomplete (Figure 3). Additionally, the arrangement of ORFs is quite similar. Furthermore, tRNA genes of these phages were also examined. All seven phages were found to possess the same four tRNA genes for methionine, tryptophan, phenylalanine, and aspartic acid (Supplementary Materials, Table S8). The eleven ORFs which were present in B1 and JA1 but absent in K (Table 6, Supplementary Materials, Figure S3) were similarly present in Team 1, vB_SauM-fRuSau02, Sb-1 and ISP. And likewise, the ORFs present in K, but absent in both B1 and JA1, were also missing in these phages. However, vB_SauM-fRuSau02 possesses a much shorter putative tail protein (RS_159) of 73 amino acids compared to the phage K counterpart (PhageK_151) of 170 amino acids. Non-hypothetical proteins that differed between these phages were a membrane protein (Phage B1_180, PhageJA1_177, and Phage_170) and an ATPase-like protein (Protein id: CCA65911.1 for phage ISP). Other ORFs that differed among these phages were mostly hypothetical proteins. 

*S. aureus* employ several defense strategies against viral attack [10,44] and these, such as restriction modification systems [45] and CRISPR-Cas systems [46], may vary from strain to strain. These defenses along with several variations encountered at the genetic level across phages B1, JA1, and K may explain the differences in host ranges observed in this study.

## 3. Materials and Methods

### 3.1. Bacterial Strains, Phage and Growth Requirement

Phages B1 and JA1 were isolated from a commercial phage cocktail purchased from the George Eliava Institute of Bacteriophage, Microbiology and Virology, Tbilisi, Georgia. The MRSA strains utilized in this study were all acquired from the Irish National MRSA Reference Laboratory, Dublin, Ireland [2] with the exception of DPC5246 and CIT281189, which are routine propagation strains utilized in our laboratory [26,36]. These strains were routinely cultured in Brain Heart Infusion broth (BHI; Sigma, St. Louis, MO, USA) at 37 °C with shaking or on BHI plates containing 1.5% (*w/v*) bacteriological agar (Sigma). All strains were stocked in BHI containing 40% glycerol and stored at −80 °C.

### 3.2. CsCl Gradient Purification

Isopycnic centrifugation through CsCl gradients was performed as previously described [47], with a number of modifications. A high titer phage lysate (>1 × 10^9^ plaque forming units [PFU] mL^−1^), was precipitated using polyethylene glycol (15% *w/v* PEG8000, 1 M NaCl) at 4 °C overnight and centrifuged, after which the pellet was resuspended in TMN buffer (10 mM Tris-HCl pH 7.4, 10 mM MgSO_4_·7H_2_O, 0.5 M NaCl). The resulting phage preparation was placed onto a CsCl step gradient composed of 1.3, 1.5, and 1.7 g/mL layers and spun in a 100 Ti rotor (Beckman Coulter, Brea, CA, USA) at 200,480 *g* for 3 h at 4 °C. The resulting phage preparations were dialyzed in Tris-HCl buffer (10 mM, pH 7.5) at 4 °C. 

### 3.3. Phage Host Range and Adsorption Study

Host range assay was performed for phages B1, JA1, and K using the plaque assay plating technique (Table 2 and Table 3). This was done in triplicate for three independent experiments. The efficiency of plaquing (EOP) was determined by dividing the phage titer on each test strain by the phage titer of the reference strain (*S. aureus* DPC5246, in the case of phages B1 and K, and *S. aureus* CIT281189 for phage JA1) [48]. An adsorption assay was performed according to the protocol previously described elsewhere with some modification [49]. Briefly, MRSA strains were grown to an optical density (OD) of 0.2 at 600 nm (estimated cell count at 10^8^ colony forming unit (cfu) mL^−1^) and 100 µL of cells were mixed with 100 µL of respective phage titered at approximately 1 × 10^7^ PFU/mL for a multiplicity of infection (MOI) of 0.1. The resulting mixtures were incubated at room temperature for 5 min to allow for phage adsorption. The bound phages were separated from the free phages by centrifugation at 14,000 rpm for 5 min. Adsorption of the phage on each strain was determined by subtracting the number of unbound phage (per mL) from the total input PFU/mL. Adsorption efficiency was expressed as a percentage relative to the propagating strain DPC5246.

### 3.4. Transmission Electron Microscopy

Electron microscopic analysis was performed following negative staining of the CsCl gradient prepared phages on freshly prepared carbon films with 2% (*w/v*) uranyl acetate. Electron micrographs were taken using a Tecnai 10 transmission electron microscope (FEI Thermo Fisher, Eindhoven, the Netherlands) at an acceleration voltage of 80 kV with a MegaView G2 CDD camera (EMSIS, Muenster, Germany).

### 3.5. Phage DNA Isolation

Phage DNA extraction was performed on CsCl purified high titer phages. These were initially treated with MgCl_2_ followed by pre-treatment with DNase and RNase for 60 min at 37 °C. Following that subsequent treatment with SDS, EDTA and proteinase K with further incubation for 60 min at 55 °C. DNA extractions were then performed on the pre-treated samples with phenol/chloroform/isoamyl alcohol (25:24:1 *v/v/v*) and chloroform/isoamyl alcohol (24:1 *v/v*). DNA precipitation was achieved using sodium acetate and 95% ethanol. DNA quality and quantity were estimated using a Nanodrop (ND-1000) and visualized following agarose gel electrophoresis

### 3.6. Phage DNA Sequencing 

DNA sequencing was performed with a high throughput Illumina HiSeq system sequencing (GATC Biotech, Konstanz, Germany). Library preparation was performed by DNA fragmentation together with adapter ligation. The libraries were then measured and quantified on a Fragment Analyzer and then sequenced to generate 2 × 300 bp paired-end reads. *De novo* assembly was performed using CLC Bio Genomics Workbench v8.0 (Aarhus, Denmark).

### 3.7. Bioinformatic Analysis

Open reading frames (ORFs) for the sequenced phages were predicted with Glimmer [50] and GenemarkS [51]. Putative functions were assigned to these ORFs using BLASTP (https://blast.ncbi.nlm.nih.gov/Blast.cgi?PAGE=Proteins), HHpred (https://toolkit.tuebingen.mpg.de/#/tools/hhpred; [52]) and InterProscan (http://www.ebi.ac.uk/interpro/search/sequence-search; [53]). Transfer RNA was predicted using tRNAscan-SE (http://lowelab.ucsc.edu/tRNAscan-SE/; [54]) and ARAGORN (http://130.235.46.10/ARAGORN/; [55]). Potential promoters were predicted using MEME (Multiple Em for Motif Elicitation) (http://meme-suite.org/tools/meme; [38]), followed by manual curation. Potential Rho-independent terminators were identified using ARNold (http://rna.igmors.u-psud.fr/toolbox/arnold; [56]) with Mfold QuikFold (http://unafold.rna.albany.edu/?q=DINAMelt/Quickfold; [57]) using RNA energy rules 3.0 to verify predictions. Artemis Comparison Tool (ACT) was used for the identification of feature variations between the genomes of phages, with homology being assessed with BLASTN [58] Genome comparison maps between phages were visualized using the Easyfig visualization tool [59]. K-like *Staphylococcus* phages used in comparative studies were K (KF766114), Team 1 (KC012913), vB_SauM-fRuSau02 (MF398190), Sb-1 (HQ163896) and ISP (FR852584).

### 3.8. Nucleotide Sequence Accession Number

The genome sequence for phages B1 and JA1 were deposited into GenBank under the accession numbers MG656408 and MF405094, respectively. 

## 4. Conclusions

Host range of three highly similar phages was performed in this study, and it was identified that phages B1 and JA1 from the Fersisi commercial phage mix had a much broader host range in comparison to phage K on a representative Irish bank of clinical MRSA sequence type isolates. Comparisons of their genomes lead to the identification of several ORFs absent in phage K, but present in both phages B1 and JA1. These ORFs were also identified in several other staphylococcal phages sourced from commercial phage mixtures (B1, JA1, Team 1 [22,23,24], vB_SauM-fRuSau02 [21], Sb-1 [19] and ISP [20]), also with a reported wide host range. The exact role of these ORFs is currently unknown. However, these ORFs along with several variations encountered at the genetic level between these phages may, in part, explain their different host range. Unfortunately, information is lacking on the influences of various phage resistance systems, which may be active in *Staphylococcus aureus*. Phage research also needs to focus more on elucidation of the functions of hypothetical proteins to allow greater understanding of how phages overcome such systems.

## Figures and Tables

**Table 1:** Dimensions of staphylococcal phages B1, JA1, and K derived from micrographs obtained from transmission electron microscopy.

Phages	Head (nm)	Tail Length (nm) (incl. “knob”)	Tail Width (nm)	Baseplate “knob” Length (nm)	Baseplate “knob” Width (nm)
B1	92.9 ± 4.0 (*n* = 11)	233.0 ± 4.4 (*n* = 12)	23.4 ± 1.2 (*n* = 12)	30.1 ± 1.8 (*n* = 12)	47.2 ± 3.7 (*n* = 10)
JA1	87.0 ± 2.1 (*n* = 9)	231.5 ± 4.7 (*n* = 9)	22.7 ± 0.9 (*n* = 9)	32.5 ± 7.9 (*n* = 9)	45.8 ± 1.4 (*n* = 9)
K	92.9 ± 3.8 (*n* = 16)	227.5 ± 5.5 (*n* = 16)	23.8 ± 1.0 (*n* = 16)	36.6 ± 5.1 (*n* = 16)	41.7 ± 2.6 (*n* = 16)

**Table 2:** Host ranges of staphylococcal phages B1, JA1, and K against methicillin-resistant *Staphylococcus aureus* (MRSA) strains from the Irish National Reference Laboratory (St. James’s Hospital Dublin, Ireland) including the efficiency of plaquing (EOP) of these strains.

*S. aureus* Strain	Phage K	Phage B1	Phage JA1
DPC5246*	1.00 ± 0.0	1.00 ± 0.0	8.98 × 10^−1^ ± 0.8
CIT281189*	No infection	No infection	1.00 ± 0.0
0.0066 (IIIV) ST239	No infection	No infection	2.59 ± 2.5
0.1206 (IV) ST250	No infection	3.89 × 10^−1^ ± 0.3	1.35 ± 1.2
0.1239 (III) ST239	No infection	1.46 × 10^−1^ ± 0.1	4.17 × 10^−2^ ± 0.0
0.1345 (II) ST5	No infection	No infection	2.08 × 10^−1^ ± 0.1
0073 (III) ST239	No infection	3.21 × 10^−1^ ± 0.2	No infection
0104 (III) ST239	No infection	3.95 × 10^−1^ ± 0.2	1.82 ± 1.6
0220 (II) ST5	3.03 × 10^−1^ ± 0.1	2.17 × 10^−1^ ± 0.2	2.38 × 10^−1^ ± 0.2
0242 (IV) ST30	4.43 × 10^−1^ ± 0.1	5.23 × 10^−1^ ± 0.5	4.90 × 10^−1^ ± 0.3
0308 (IA) ST247	1.40 ± 0.2	1.36 ± 1.3	1.71 ± 1.6
3045 (IIV) ST8	No infection	4.93 × 10^−2^ ± 0.0	1.69 ± 0.7
3144 (IIV) ST8	No infection	1.21 ± 1.0	2.17 ± 1.2
3488 (VV) ST8	No infection	No infection	No infection
3581 (IA) ST247	No infection	No infection	9.26 × 10^−1^ ± 0.7
3594 (II) ST36	4.38 × 10^−1^ ± 0.1	8.67 × 10^−1^ ± 0.4	1.06 ± 0.7
3596 (IIV) ST8	2.49 × 10^−4^ ± 0.0	1.29 ± 0.9	3.59 ± 2.7
E1038 (IIV) ST8	1.27 × 10^−4^ ± 0.0	2.02 × 10^−1^ ± 0.2	1.89 ± 1.4
E1139 (IV) ST45	No infection	3.88 × 10^−6^ ± 0.0	No infection
E1174 (IV) ST22	7.03 × 10^−1^ ± 0.7	3.11 × 10^−1^ ± 0.2	No infection
E1185 (IV) ST12	1.16 × 10^−6^ ± 0.0	No infection	No infection
E1202 (II) ST496	No infection	4.79 × 10^−1^ ± 0.2	9.49 × 10^−1^ ± 0.8
M03/0073 (III) ST239	1.76 ± 0.5	1.51 ± 0.8	2.30 ± 0.7

* *S. aureus* strains for phage propagation; data is represented as means ± standard deviations based on triplicate measurements.

**Table 3:** Zone sizes and morphologies of B1, JA1, and K plaques formed on MRSA strains collected from the Irish National MRSA Reference Laboratory (St. James’s Hospital Dublin, Ireland).

*S. aureus* Strain	Phage K	Phage B1	Phage JA1
DPC5246	2 mm	1 mm with halo to 2 mm	1 mm with halo to 2 mm
CIT281189	No plaques	No plaques	1.5 mm
0.0066 (IIV) ST239	No plaques	No plaques	1 mm
0.1206 (IV) ST250	No plaques	2 mm	0.5 mm with halo to 1 mm
0.1239 (III) ST239	No plaques	0.5 mm, faint plaques	1 mm
0.1345 (II) ST5	No plaques	No plaques	1 mm
0073 (III) ST239	No plaques	0.5 mm	No plaques
0104 (III) ST239	No plaques	0.5 mm	1 mm
0220 (II) ST5	0.5 mm	1 mm	1 mm
0242 (IV) ST30	1 mm	1.5 mm	1.5 mm
0308 (IA) ST247	1 mm	1 mm	0.5 mm, faint plaques
3045 (IIV) ST8	No plaques	1 mm	1 mm
3144 (IIV) ST8	No plaques	1.5 mm, faint plaques	1 mm
3488 (VV) ST8	No plaques	0.5 mm, faint plaques	0.5 mm with halo to 1 mm
3581 (IA) ST247	No plaques	No plaques	1 mm
3594 (II) ST36	1.5 mm	1 mm	1.5 mm
3596 (IIV) ST8	0.5 mm	0.5 mm with halo to 1.5 mm	0.5 mm with halo to 1.5 mm
E1038 (IIV) ST8	0.5 mm, faint plaques	0.5 mm, faint plaques	1.5 mm
E1139 (IV) ST45	No plaques	0.5 mm, faint plaques	No plaques
E1174 (IV) ST22	0.5 mm, faint plaques	0.5 mm	No plaques
E1185 (IV) ST12	0.5 mm, faint plaques	No plaques	No plaques
E1202 (II) ST496	No plaques	1 mm	0.5 mm
M03/0073 (III) ST239	2 mm	0.5 mm with halo to 1.5 mm	0.5 mm with halo to 1.5 mm

**Table 4:** List of missing ORFs present in phage B1 but absent in phage JA1.

ORFs	Amino Acid Numbers	Protein Size (kDa)	Predicted Function
PhageB1_009	112	13.5	Terminal repeat encoded protein
PhageB1_016	107	12.4	Terminal repeat encoded protein
PhageB1_202	32	3.5	Unknown
PhageB1_203	104	11.6	Unknown

**Table 5:** List of missing ORFs and their predicted putative functions absent in both phages B1 and JA1 but present in phage K.

ORFs	Amino Acid Number	Protein Size (kDa)	Predicted Function
PhageK_004	108	12.7	Unknown
PhageK_016*	107	12.4	Unknown
PhageK_019	57	4.7	Unknown
PhageK_020	89	10.2	Unknown
PhageK_168	185	21.7	Predicted to contain a transmembrane region based on InterProScan
PhageK_187	101	11.7	Unknown
PhageK_188	123	13.8	Predicted to contain a transmembrane region based on InterProScan
PhageK_189	78	9.2	Unknown
PhageK_190	175	20.6	Predicted as a putative metallophoshatase
PhageK_191	106	12.9	Unknown
PhageK_192	76	8.9	Predicted to contain a transmembrane region based on InterProScan
PhageK_196	226	25.8	Unknown
PhageK_205	83	9.7	Unknown
PhageK_206	98	11.2	Unknown
PhageK_208	99	11.6	Unknown
PhageK_209	75	8.9	Unknown
PhageK_211	117	13.9	Predicted to possess a transmembrane region based on InterProScan
PhageK_212	128	15.6	Unknown

* ORF that phage JA1 does not share with phage K.

**Table 6:** List of missing ORFs and their predicted function absent in phage K but present in phages B1 and JA1.

ORFs	Amino Acid Number	Protein Size (kDa)	Predicted Function
PhageJA1_003 (PhageB1_003)	96	11.3	Unknown
PhageJA1_020 (PhageB1_022)	161	19.1	Unknown
PhageJA1_021 (PhageB1_023)	135	16.5	Unknown
PhageJA1_084 (PhageB1_087)	323	39.6	Predicted as a putative endonuclease interrupting the terminase large subunit [PhageJA1_083 (PhageB1_086) and PhageJA1_085 (PhageB1_088)]
PhageJA1_152 (PhageB1_155)	322	38.3	Predicted as a putative endonuclease containing a LAGLIDADG-like domain and an Intein splicing domain and interrupts the DNA repair protein [PhageJA1_151 (PhageB1_154) and PhageJA1_153 (PhageB1_156)]
PhageJA1_206 (PhageB1_212)	73	8.9	Unknown
PhageJA1_208 (PhageB1_214)	169	20.3	HHpred indicates homology to cell wall hydrolases
PhageJA1_209 (PhageB1_215)	109	12.6	Unknown
PhageJA1_211 (PhageB1_217)	104	12.0	Unknown
PhageJA1_212 (PhageB1_218)	55	6.5	Unknown
PhageJA1_213 (PhageB1_219)	33	3.7	Predicted to possess a transmembrane region based on InterProScan

## Supplementary Materials

The following are available online at http://www.mdpi.com/2079-6382/7/2/37/s1, Figure S1: Plaque morphologies of phages B1, JA1 and K with common morphology types encountered in their host range study to include plaques sizes of 2mm (A), 0.5mm (B) and 1.0mm (C), Figure S2: *Staphylococcus* phage K adsorption to strains of *Staphylococcus aureus* resistant to infection by, in comparison host strain DPC5246, Figure S3: Comparison of regions within the genome of phage K to closely related staphylococcal phages, Table S1: Annotation of the staphylococcal phage vB_SauM_B1, Table S2: Annotation of the staphylococcal phage vB_SauM_JA1, Table S3: Predicted Rho-like promoters of *Staphylococcus* phage B1 genome found using MEME, Table S4: Predicted Rho-like promoters of *Staphylococcus* phage JA1 genome found using MEME, Table S5: High ΔG rho-independent terminators predicted in the genome *Staphylococcus* phage B1 identified using ARNold and QuikFold, Table S6: High ΔG rho-independent terminators predicted in the genome *Staphylococcus* phage JA1 identified using ARNold and QuikFold, Table S7: Percentage similarity based on BLASTN of broad host range *Staphylococcus* phages that form commercial phage cocktails to that of *Staphylococcus* phage K, Table S8: tRNA genes of phages B1, JA1, K, vB_SauM-fRuSau02, ISP, Sb-1, Team 1.
