# Supplementary material for: Comparison of Staphylococcus Phage K with Close Phage Relatives Commonly Employed in Phage Therapeutics
Source: Antibiotics (Basel). 2018 Apr 25;7(2):37. doi: 10.3390/antibiotics7020037 (PMC6022877; doi:10.3390/antibiotics7020037)
Supplement: Supplementary file 1 [file antibiotics-07-00037-s001.zip › Ajuebor et al. supplementary material Table S3-S7, Figure S1-S3_final corrections.docx]

**Table S3**. Predicted Rho-like promoters of *Staphylococcus* phage B1 found using MEME

| no. | Promoter | start | stop | -35 | spacer | -10 |
| --- | --- | --- | --- | --- | --- | --- |
| 1 | B1P_13 | 5,687 | 5,659 | TTGACA | TTAAGACCGAATTATTA | TATAAT |
| 2 | B1P_14 | 5,733 | 5,761 | TTGACT | TTAATATCATTATAGTT | TAATAT |
| 3 | B1P_15 | 5,956 | 5,984 | TTGACA | ACCTAGAAACAACATGT | TAATAT |
| 4 | B1P_16 | 6,253 | 6,281 | TTGACA | GTCACTTGAAACCATGA | TATTAT |
| 5 | B1P_17 | 6,658 | 6,686 | TTGACT | TTCAAGCCCTACAATGT | TATTAT |
| 6 | B1P_18 | 6,989 | 7,017 | TTGACA | TCCTAACATATAGATGG | TAATAT |
| 7 | B1P_31 | 13,184 | 13,156 | TTGACT | TTTTTTACTAAGTATGG | TAAGAT |
| 8 | B1P_37 | 16,852 | 16,824 | TTGACA | TTATTATCAATATATGT | TATTAT |
| 9 | B1P_40 | 17,662 | 17,634 | TTGACA | AAATATAAAAAATAGTG | TATAGT |
| 10 | B1P_41 | 17,937 | 17,909 | ATGACT | TAGAAAAAGACCTATGA | TATATT |
| 11 | B1P_48 | 22,004 | 21,976 | TTGACA | AATACAAATACTTGTAA | TATAAT |
| 12 | B1P_54 | 25,768 | 25,740 | TTGACA | AATATTATTTACTATGG | TATGAT |
| 13 | B1P_52 | 23,499 | 23,471 | TTGACA | ATAGTATCATAATATGA | TATAAT |
| 14 | B1P_60 | 28,616 | 28,588 | TTGACA | AATCCCCTTAGTTATGG | TATAAT |
| 15 | B1P_64 | 30,702 | 30,674 | TTGAGT | TAGTTATTAATTTAAAA | TAAAAT |
| 16 | B1P_67 | 31,829 | 31,801 | TTGACT | TCATAAGTTAACTATGC | TATAAT |
| 17 | B1P_69 | 33,442 | 33,414 | TTGACA | TAGGTGGTTTTTTATGC | TATAGT |
| 18 | B1P_68 | 32,658 | 32,630 | TTGCGT | TATTTAAAGATATATGT | TATGAT |
| 19 | B1P_71 | 34,618 | 34,590 | TTGACA | AAATTAAATACATAGTG | TATAGT |
| 20 | B1P_74 | 36,120 | 36,092 | TTGACA | ACATAATAACTTTCCTA | TATACT |
| 21 | B1P_78 | 39,639 | 39,611 | TTGACT | TATTTATCAATATAGTA | TATAGT |
| 22 | B1P_107 | 60,449 | 60,477 | TTGACA | CTTTAAAATTTATATGT | TATTAT |
| 23 | B1P_108 | 60,634 | 60,662 | TTGACA | ATTATAATTAACTAAGG | TATATT |
| 24 | B1P_109 | 61,137 | 61,165 | TTGACA | ATTCAATAAGGAGGTAT | TATAAT |
| 25 | B1P_110 | 61,378 | 61,406 | TTGACA | AATTAAAACTAATAAAT | TATAAT |
| 26 | B1P_114 | 67,043 | 67,071 | TTGACA | CAAGAGTAGTATCATAA | TATACT |
| 27 | B1P_128 | 87,304 | 87,332 | TTGACT | TGAAAAGGATTCTGTGG | TATACT |
| 28 | B1P_131 | 92,191 | 92,219 | TTGACA | TTTTATATGTTAGGTGG | TATAAT |
| 29 | B1P_136 | 97,265 | 97,293 | TTGACC | TTAGAGAAGTTTTATGT | TATACT |
| 30 | B1P_146 | 104,101 | 104,129 | TTGACA | AGGTTTAAAATATATGG | TATAGT |
| 31 | B1P_151 | 109,267 | 109,295 | TTGACA | ATATAGTTAACTTATGT | TATACT |
| 32 | B1P_153 | 110,090 | 110,118 | TCATAA | ATATAAAAAACTATGT | TATAAT |
| 33 | B1P_162 | 116,697 | 116,725 | TTGACA | ATTTATAATATCTATGA | TACACT |
| 34 | B1P_167 | 119,714 | 119,742 | TTGACT | CTTTTTACTATATATGG | TATATT |
| 35 | B1P_173 | 123,928 | 123,956 | TTGACA | GCTCCTATAGTTTATGA | TATAGT |
| 36 | B1P_176 | 125,707 | 125,735 | TTGACT | CTCTTTTTGTTTTATGG | TATATT |
| 37 | B1P_179 | 126,322 | 126,350 | TTGACA | AGAACAAATAAGTGTAG | TATAGT |
| 38 | B1P_184 | 127,793 | 127,821 | TTGACA | GATGAAGCATTTTAATA | TATACT |
| 39 | B1P_188 | 129,072 | 129,100 | TTGACA | CCTTTGTACTTTTGTAT | TATACT |
| 40 | B1P_193 | 130,673 | 130,701 | TTGACA | ATTGAGTATACATAGGT | TATACT |
| 41 | B1P_199 | 132,225 | 132,253 | TTGACA | TTAGGTTTCTTTTATTA | TATACT |
| 42 | B1P_204 | 133,772 | 133,800 | TTGACA | GCAGGTATTTTTTATAG | TATACT |
| 43 | B1P_210 | 137,326 | 137,354 | TTGACA | AAGGGAGTTTTTTATTA | TATAGT |
| 44 | B1P_213 | 138,281 | 138,309 | TTGACT | TAGGTAGGTATCTATTA | TATAAT |

**Table S4.** Predicted Rho-like promoters of *Staphylococcus* phage JA1 found using MEME.

| no. | Promoter | start | stop | -35 | spacer | -10 |
| --- | --- | --- | --- | --- | --- | --- |
| 1 | JA1P_12 | 5,657 | 5,685 | TTGACA | TTAAGACCGAATTATTA | TATAAT |
| 2 | JA1P_13 | 5,731 | 5,759 | TTGACT | TTAATATCATTATAGTT | TAATAT |
| 3 | JA1P_14 | 5,954 | 5,982 | TTGACA | ACCTAGAAACAACATGT | TAATAT |
| 4 | JA1P_15 | 6,251 | 6,279 | TTGACA | GTCACTTGAAACCATGA | TATTAT |
| 5 | JA1P_16 | 6,564 | 6,592 | TTGACA | TCCTAACATATAGATGG | TAATAT |
| 6 | JA1P_29 | 12,759 | 12,731 | TTGACT | TTTTTTACTAAGTATGG | TAAGAT |
| 7 | JA1P_35 | 16427 | 16,399 | TTGACA | TTATTATCAATATATGT | TATTAT |
| 8 | JA1P_38 | 17,237 | 17,209 | TTGACA | AAATATAAAAAATAGTG | TATAGT |
| 9 | JA1P_39 | 17,512 | 17,484 | ATGACT | TAGAAAAAGACCTATGA | TATATT |
| 10 | JA1P_46 | 21,579 | 21,551 | TTGACA | AATACAAATACTTGTAA | TATAAT |
| 11 | JA1P_50 | 23,072 | 23,044 | TTGACA | ATAGTATCATAATATGA | TATAAT |
| 12 | PJA1_52 | 25,341 | 25,313 | TTGACA | AATATTATTTACTATGG | TATGAT |
| 13 | JA1P_58 | 28,189 | 28,161 | TTGACA | AATCACCTTAGTTATGG | TATAAT |
| 14 | JA1P_62 | 30,275 | 30,247 | TTGAGT | TAGTTATTAATTTAAAA | TAAAAT |
| 15 | JA1P_65 | 31,402 | 31,374 | TTGACT | TCATAAGTTAACTATGC | TATAAT |
| 16 | JA1P_66 | 32,231 | 32,203 | TTGCGT | TATTTAAAGATATATGT | TATGAT |
| 17 | JA1P_69 | 34,191 | 34,163 | TTGACA | AAATTAAATACATAGTG | TATAGT |
| 18 | JA1P_67 | 33,015 | 32,987 | TTGACA | TAGGTGGTTTTTTATGC | TATAGT |
| 19 | JA1P_71 | 35,693 | 35,665 | TTGACA | ACATAATAACTTTCCTA | TATACT |
| 20 | JA1P_75 | 39,212 | 39,184 | TTGACT | TATTTATCAATATAGTA | TATAGT |
| 21 | JA1P_105 | 60,202 | 60,230 | TTGACA | ATTATAATTAACTAAGG | TATATT |
| 22 | JA1P_198 | 132,737 | 132,765 | TTGACA | GCAGGTATTTTTTATAG | TATACT |
| 23 | JA1P_204 | 136,291 | 136,319 | TTGACA | AAGGGAGTTTTTTATTA | TATAGT |
| 24 | JA1P_104 | 60,017 | 60,045 | TTGACA | CTTTAAAATTTATATGT | TATTAT |
| 25 | JA1P_106 | 60,705 | 60,733 | TTGACA | ATTCAATAAGGAGGTAT | TATAAT |
| 26 | JA1P_107 | 60,946 | 60,974 | TTGACA | AATTAAAACTAATAAAT | TATAAT |
| 27 | JA1P_111 | 66,611 | 66,639 | TTGACA | CAAGAGTAGTATCATAA | TATACT |
| 28 | JA1P_125 | 86,872 | 86,900 | TTGACT | TGAAAAGGATTCTGTGG | TATACT |
| 29 | JA1P_127 | 91,759 | 91,787 | TTGACA | TTTTATATGTTAGGTGG | TATAAT |
| 30 | JA1P_133 | 96,833 | 96,861 | TTGACC | TTAGAGAAGTTTTATGT | TATACT |
| 31 | JA1P_143 | 103,669 | 103,697 | TTGACA | AGGTTTAAAATATATGG | TATAGT |
| 32 | JA1P_148 | 108,835 | 108,863 | TTGACA | ATATAGTTAACTTATGT | TATACT |
| 33 | JA1P_150 | 109,658 | 109,686 | TTGACA | AATATAAAAAACTATGT | TATAAT |
| 34 | JA1P_159 | 116,256 | 116,284 | TTGACA | ATTTATAATATCTATGA | TACACT |
| 35 | JA1P_164 | 119,273 | 119,301 | TTGACT | CTTTTTACTATATATGG | TATATT |
| 36 | JA1P_170 | 123,487 | 123,515 | TTGACA | GCTCCTATAGTTTATGA | TATAGT |
| 37 | JA1P_173 | 125,266 | 125,294 | TTGACT | CTCTTTTTGTTTTATGG | TATATT |
| 38 | JA1P_176 | 125,881 | 125,909 | TTGACA | AGAACAAATAAGTGTAG | TATAGT |
| 39 | JA1P_181 | 127,352 | 127,380 | TTGACA | GATGAAGCATTTTAATA | TATACT |
| 40 | JA1P_185 | 128,636 | 128,665 | TTGACA | CCTTTGTACTTTTGTAT | TATACT |
| 41 | JA1P_189 | 130,237 | 130,265 | TTGACA | ATTGAGTATACATAAGT | TATACT |
| 42 | JA1P_195 | 141,789 | 131,817 | TTGACA | TTAGGTTTCTTTTATTA | TATACT |
| 43 | JA1P_207 | 137,246 | 137,274 | TTGACT | TAGGTAGGTATCTATTA | TATAAT |

**Table S5**. High ΔG rho-independent terminators predicted in the genome Staphylococcus phage B1 identified using ARNold and QuikFold

| no. | Terminator | Coordinates | Sequence | ΔG kcal/mol |
| --- | --- | --- | --- | --- |
| 1 | B1T_9 | 3056-3093 | ACACTAGGAATAATATCCTAGTGTaTTTATTTTTGCGG | -12.8 |
| 2 | B1T_8 | 3071-3104 | CACTAGGATATTATTCCTAGTGTATTATATAATT | -11.8 |
| 3 | B1T_12 | 4828-4865 | TCCCTAGAAATCTAATCCTAGGGAaTTGTATAATTTTT | -9.8 |
| 4 | B1T_13 | 4828-4865 | TCCCTAGGATTAGATTTCTAGGGATTTTTATTTATT | -13.1 |
| 5 | B1T_13a | 5137-5103 | AGAAAAGGGTTGACCTTTTCTtTTTTCTATAGTAT | -9 |
| 6 | B1T_20 | 7960-7989 | GAGGGAATAAAATCCCTCTTTTATTTTTAT | -9.6 |
| 7 | B1T_21 | 8280-8247 | GGAGGGATTTAATTTCCCTCTTTTTTTATTTTAG | -10.4 |
| 8 | B1T_41 | 17691-17657 | AGGCTACTTTAATTAGTAGCCTTTTTTTGTTGACA | -11.5 |
| 9 | B1T_43 | 18458-18425 | GCAGACTTTTAATAAGTCTGCTTTTCTCTTATAT | -11.6 |
| 10 | B1T_51 | 22889-22852 | CACCTTGCTTGTAGCCAAGCAGGGTGTTTTTTTTTTAT | -16.9 |
| 11 | B1T_68 | 31869-31834 | GACTAAGATTAATTTCTTAGTCtTTTTTTGTATATT | -10.3 |
| 12 | B1T_70 | 33449-33417 | CCACCTATTGACATAGGTGGTTTTTTATGCTAT | -10.5 |
| 13 | B1T_72 | 34659-34625 | AGACGGATTTTAAATCCGTCTaTTTTTTTTGCAAA | -10.8 |
| 14 | B1T_92 | 48262-48291 | GAGGAGTAATTACTCCTCTTTTTTGTTTGC | -10.6 |
| 15 | B1T_95 | 50787-50820 | AGCCTAGAATAAATCTAGGCTTTGTTTATTTTTT | -11 |
| 16 | B1T_98 | 54261-54296 | TAGGGTACAGTAAAATGTACCCTATTTATATTCTTT | -12.8 |
| 17 | B1T_106 | 60421-60452 | GACCAACTAAAAAGTTGGTCTTTTTTTATTGA | -11.3 |
| 18 | B1T_112 | 62923-62958 | GGGTGGTAGGTGATACTACCATCCTTATTTTTTTAA | -15.4 |
| 19 | B1T_116 | 62923-62958 | AGACCTATTAATTTAGGTCTTTTTTTAGTTGTA | -8.7 |
| 20 | B1T_123 | 83367-83398 | GAGGGGTTGATTGACCCCTCTTTATTTAATAA | -14.2 |
| 21 | B1T_127 | 87272-87305 | GACTAGGAGAAATTTCCTAGTCTTTTTTTTTCTT | -12.3 |
| 22 | B1T_140 | 101361-10139 | TTGGGAGCAAGGAATCTCCCAATTTTGGACTCCT | -9.1 |
| 23 | B1T_145 | 104073-104106 | GAAGAGAAATAATTCTCTTCtTTTTTTATTGACA | -9.1 |
| 24 | B1T_153 | 111422-111462 | GAGTGCCTTAGAGCACTCTTTTATTTGAGA | -9 |
| 25 | B1T_161 | 116669-116700 | GACCAACTAAAAAGTTGGTCTTTTTTTATTGA | -11.3 |
| 26 | B1T_166 | 119699-119732 | GAGTCAAGTCTTTACTTGACTCTTTTTACTATAT | -12 |
| 27 | B1T_175 | 125692-125725 | GAGTCAAGTTAATTCTTGACTCTCTTTTTGTTTT | -11.5 |
| 28 | B1T_181 | 127410-127446 | GAAGGTAGAGAATAAGCTACCTTCTTCTACTCCTATT | -11.2 |
| 29 | B1T_203 | 133755-133797 | TACCTGTTGACAGCCTGTTGACAGCAGGTATTTTTTATAGTAT | -14.1 |
| 30 | B1T_209 | 137317-137354 | AACTCCCTATTGACAAAGGGAGTTtTTTATTATATAGT | -10.8 |

**Table S6**. High ΔG rho-independent terminators predicted in the genome Staphylococcus phage JA1 identified using ARNold and QuikFold

| Terminator | Coordinates | Sequence | ΔG kcal/mol |
| --- | --- | --- | --- |
| JA1T_8 | 3069-3102 | CACTAGGATATTATTCCTAGTGTATTATATAATT | -11.8 |
| JA1T_11 | 4840-4875 | TCCCTAGGATTAGATTTCTAGGGATTTTTATTTATT | -13.1 |
| JA1T_12 | 5135-5101 | AGAAAAGGGTTGACCTTTTCTtTTTTCTATAGTAT | -9 |
| JA1T_18 | 7535-7564 | GAGGGAATAAAATCCCTCTTTTATTTTTAT | -9.6 |
| JA1T_19 | 7855-7822 | GGAGGGATTTAATTTCCCTCTTTTTTTATTTTAG | -10.4 |
| JA1T_30 | 12789-12754 | ACACCTATTAATTTAATAGGTGTTTTTTTATTGACT | -9.9 |
| JA1T_41 | 18033-18000 | GCAGACTTTTAATAAGTCTGCTTTTCTCTTATAT | -11.6 |
| JA1T_47 | 21654-21611 | TACCTTACCCTATGTTAAGTTATAGGTGTAAGGTATTTTTTTTT | -17.4 |
| JA1T_49 | 22462-22425 | CACCTTGCTTGTAGCCAAGCAGGGTGTTTTTTTTATAT | -16.9 |
| JA1T_51 | 25371-25338 | GAAGGACTTTAAAAAGTTCTTCTTTTTTTGTTGA | -9.3 |
| JA1T_66 | 31442-31407 | GACTAAGATTAATTTCTTAGTCtTTTTTTGTATATT | -9.3 |
| JA1T_68 | 33022-31990 | CCACCTATTGACATAGGTGGTTTTTTATGCTAT | -10.5 |
| JA1T_70 | 34232-32198 | AGACGGATTTTAAATCCGTCTaTTTTTTTTGCAAA | -10.8 |
| JA1T_89 | 47829-47858 | GAGGAGTAATTACTCCTCTTTTTTTGTTTG | -10.6 |
| JA1T_92 | 48657-50348 | AGCCTAGAATAAATCTAGGCTTTGTTTATTTTTT | -11 |
| JA1T_95 | 53829-53864 | GGGATAAACTTAGGGTTTATCCCTTTTTTATTAAAA | -12.8 |
| JA1T_103 | 53829-53864 | GACCAACTAAAAAGTTGGTCTTTTTTTATTGA | -11.3 |
| JA1T_109 | 62491-62526 | GGGTGGTAGGTGATACTACCATCCTTATTTTTTTAA | -15.4 |
| JA1T_113 | 72559-72591 | AGACCTATTAATTTAGGTCTTTTTTTAGTTGTA | -8.7 |
| JA1T_120 | 82935-82966 | GAGGGGTTGATTGACCCCTCTTTATTTAATAA | -14.2 |
| JA1T_124 | 86840-86873 | GACTAGGAGAAATTTCCTAGTCTTTTTTTTTCTT | -12.3 |
| JA1T_137 | 100929-100962 | TTGGGAGCAAGGAATCTCCCAATTTTGGACTCCT | -9.1 |
| JA1T_142 | 103641-103674 | GAAGAGAAATAATTCTCTTCtTTTTTTATTGACA | -9.1 |
| JA1T_150 | 111001-111030 | GAGTGCCTTAGAGCACTCTTTTATTTGAGA | -9 |
| JA1T_158 | 116228-116259 | GACCAACTAAAAAGTTGGTCTTTTTTTATTGA | -11.3 |
| JA1T_163 | 119258-119291 | GAGTCAAGTCTTTACTTGACTCTTTTTACTATAT | -12 |
| JA1T_172 | 125251-125284 | GAGTCAAGTTAATTCTTGACTCTCTTTTTGTTTT | -11.5 |
| JA1T_178 | 126969-127005 | GAAGGTAGAGAATAAGCTACCTTCTTCTACTCCTATT | -11.2 |
| JA1T_197 | 132720-132762 | TACCTGTTGACAGCCTGTTGACAGCAGGTATTTTTTATAGTAT | -14.2 |
| JA1T_203 | 136282-136319 | AACTCCCTATTGACAAAGGGAGTTtTTTATTATATAGT | -10.8 |

**Table S7**: Percentage similarity based on BLASTN of broad host range *Staphylococcus* phages that form commercial phage cocktails to that of *Staphylococcus* phage K

| Phage | Accession | Identity vs phage K, % |
| --- | --- | --- |
| Phage K | KF766114 | 100 |
| Team 1 | KC012913 | 95 |
| fRuSau02 | MF398190 | 95 |
| Sb-1 | HQ163896 | 85 |
| ISP | FR852584 | 96 |

**Figure S1**. Plaque morphologies of phages B1, JA1 and K with common morphology types encountered in their host range study to include plaques sizes of 2mm (A), 0.5mm (B) and 1.0mm (C). Plaque morphologies with halos were encountered but were not clearly seen as photographs. Faint plaques were also encountered and these could only be clearly seen in direct path of light (C)

**Figure S2**. *Staphylococcus* phage K adsorption to strains of *Staphylococcus aureus* resistant to infection by, in comparison host strain DPC5246

**Figure S3.** Comparison of regions within the genome of phage K to closely related staphylococcal phages (B1, JA1, Team 1, fRuSau02, Sb-1 and ISP) commonly employed as commercial phage mixtures using currently available annotations employing BLASTN and visualized with Easyfig. Several ORFs absent in phage K but present in both B1 and JA1 [phageJA1_020 & phageJA1_021 (A) and phage_206, phageJA1_208, phageJA1_209, phageJA1_211, phageJA1_212 & phageJA1_213 (B)] were also encountered in these closely related staphylococcal phages with reported wide host range.
